# Supplementary material for: A distinct growth physiology enhances bacterial growth under rapid nutrient fluctuations
Source: Nat Commun. 2021 Jun 16;12:3662. doi: 10.1038/s41467-021-23439-8 (PMC8209047; doi:10.1038/s41467-021-23439-8)
Supplement: Supplementary file 1 — Supplementary Information [file 41467_2021_23439_MOESM1_ESM.pdf]

Supplementary Information

A distinct growth physiology enhances bacterial growth under rapid nutrient fluctuations

Nguyen *et al.*

Supplementary Fig. 1: The Microfluidic Signal Generator (MSG) delivers fluctuating nutrient signals alongside steady ones.

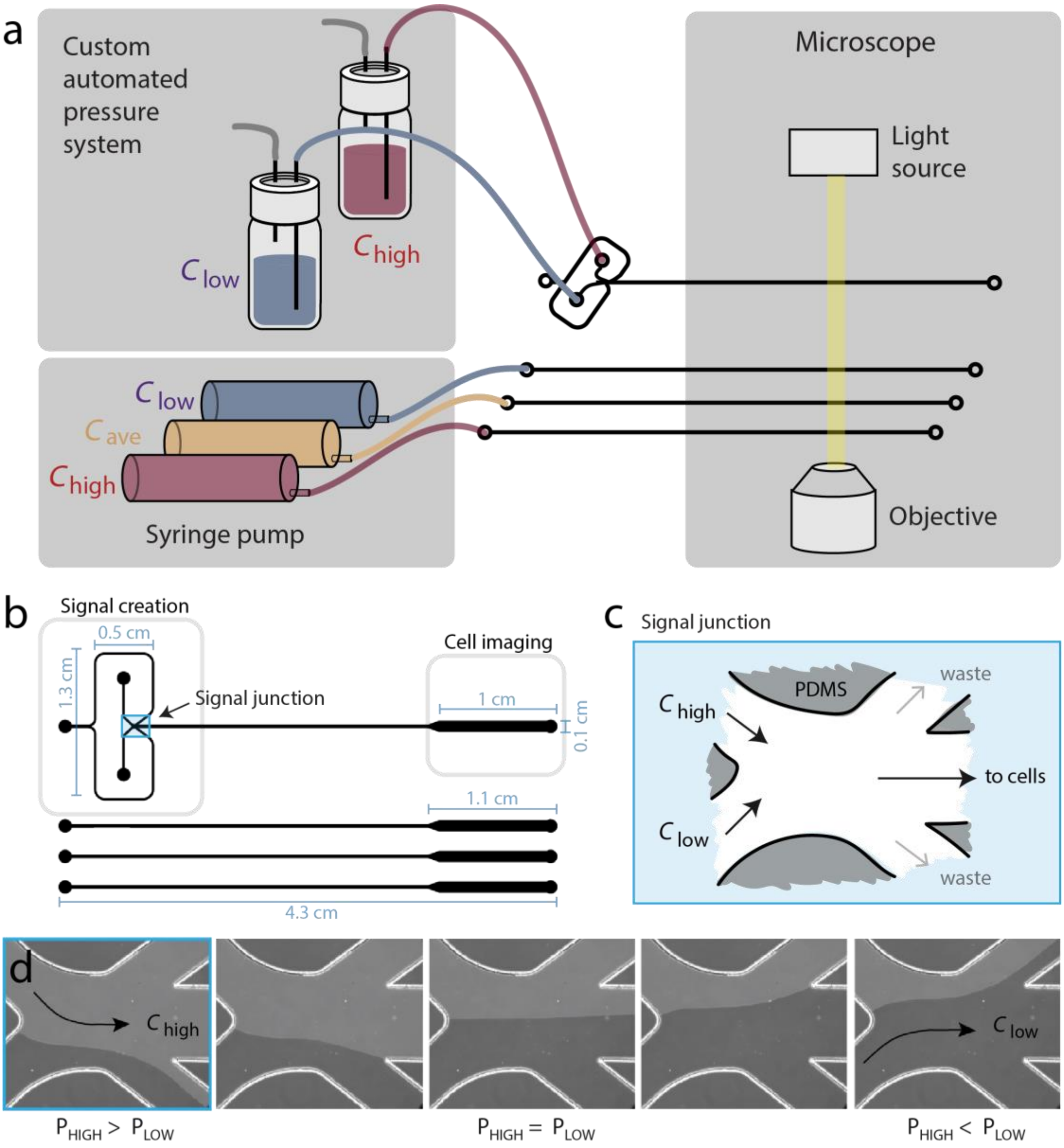

a Four nutrient signals are delivered within each experiment, in parallel microfluidic channels imaged by the microscope. The fluctuating signal is produced by oscillating the pressure in two media vials, one containing the high nutrient concentration (C_{high}) and one containing the low concentration (C_{low}), at a user-programmed frequency. The programmed signal automates which vial has the higher internal pressure, and the system is calibrated such that, when the pressure in the C_{high} vial is higher, only C_{high} reaches the cells downstream. The fluid input to each steady environment is a single syringe filled with either C_{low} (purple), C_{ave} (gold) or C_{high} (red). All three syringes are pushed by the same syringe pump at a flow rate of 15 µL/min. In the diagram, the lines (colored by nutrient concentration) represent the flexible polyethylene tubing connecting each fluid input with the microchannel inlets. The circular

microchannel features not associated with inputs are the microchannel outlets, which are connected via tubing to waste receptacles (not shown). **b** 2-D design of microfluidic device. The channel height (not shown) is uniformly 60 µm. The top channel with the unique upstream feature is the MSG, and the three straight channels deliver the control steady environments. In all channels, the nutrient signal travels over 2.5 cm before reaching the cells. **c** The signal junction upstream in the MSG has two inlets and three outlets: one inlet per medium (C_{low} and C_{high}), one outlet towards the cells, and two waste outlets to remove excess fluid that does not enter the middle channel. **d** Nutrient switches are achieved via pressure oscillations in the switching junction. When the pressure in the low nutrient reservoir is approximately equal to that in the high nutrient reservoir, then both media flow to the cells downstream. To generate square waves that oscillate between C_{low} and C_{high}, we calibrated the pressure ratios required such that only C_{low} or only C_{high} would enter the middle outlet toward the cells.

Supplementary Fig. 2: Control experiments demonstrate nutrient concentration is the determinant of growth rate.

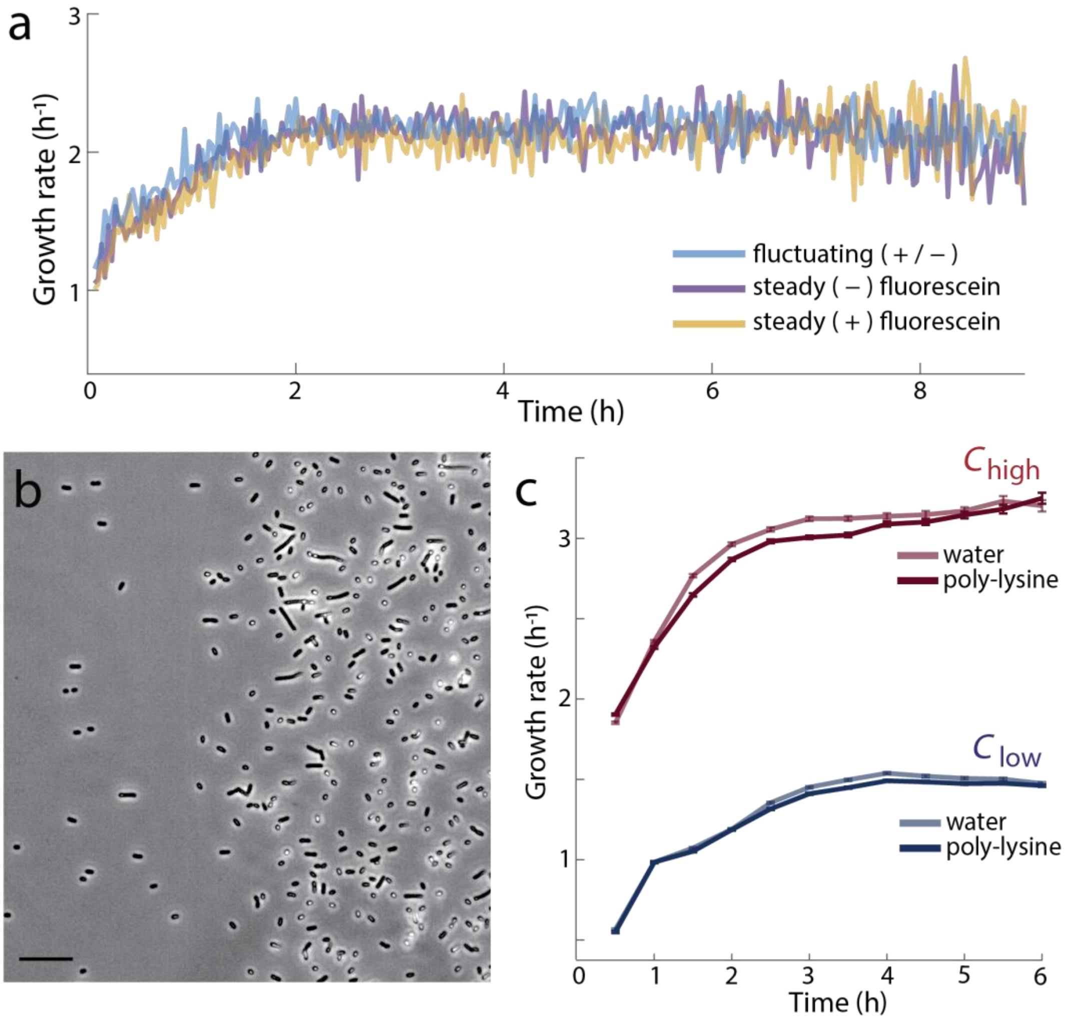

a Neither fluorescein labeling nor flow rate affect growth rate. Time-evolution of growth rate in three parallel channels: (1) fluctuations between fluorescein-labeled (0.26 nM) and unlabeled nutrient on a 30 s period (blue), (2) steady nutrient without fluorescein (purple), and (3) steady nutrient with 0.26 nM fluorescein (yellow). All nutrient media are 1% LB diluted in equimolar salt solution, approximately equal to C_{ave} (1.05% LB). Flow rate within the fluctuating channel is 22 μL min^{-1}, as generated by the compressed air-based pressure system. Flow rate within the steady channels is 15 μL min^{-1} generated by a syringe pump. Each curve represents the mean instantaneous growth rate for each condition. **b** Poly-L-lysine treatment enhances bacterial attachment. A phase contrast image of a microchannel with *E. coli* attached to the lower, glass surface in flow. The right-hand side of the image was treated with poly-lysine prior to inoculation while the left-hand side was not. The boundary of the poly-lysine treatment is clearly marked by the increased presence of adherent cells. Scale bar denotes 10 μm. **c** Nutrient, not poly-L-lysine, determines growth rate. Mean instantaneous growth rate over time under steady nutrient conditions demonstrates that

cells surface-attached to a poly-L-lysine-treated surface display no difference in growth rate compared to cells surface-attached to an untreated glass surface. Two channels delivered C_{high} (red) and two channels delivered C_{low} (navy), of which one of each was treated with poly-L-lysine (darker line) and the other with sterile milliQ water (lighter line).

Supplementary Fig. 3: Media switching occurs within 3 s at user-defined frequencies.

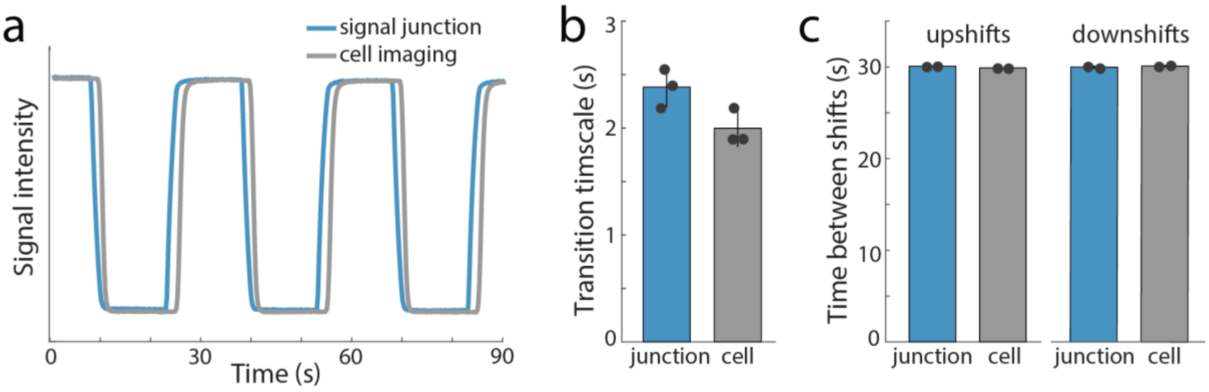

a Fluctuations between two media, one of which was labeled with 0.26 nM sodium fluorescein, at the signal junction and near the end of the cell-imaging region of the MSG. A switch is considered complete when the fluorescence intensity reaches the baseline or the saturated intensity. **b** Measured transition time at the signal junction and the end of the cell-imaging region. Transition time is defined as the time required to fully switch between media. Error bars are standard deviation of the mean, measured from *n* = 3 transitions each. Individual measurements are overlaid as dark gray data points. **c** Period lengths measured from the fluorescent signal, which was programmed to oscillate on a 30 s period, demonstrate that the periodic oscillations are robust. Upshifts measure the time between peak fluorescent signals. Downshifts measure the time between troughs. The programmed period was reliably quantified between peaks and between troughs from the repetitive fluorescein signal. Individual measurements are overlaid as dark gray data points. **a-c** The analyses shown are from one representative of *n* = 3 independent experiments.

Supplementary Fig. 4: Growth rate does not vary with distance from the signal junction.

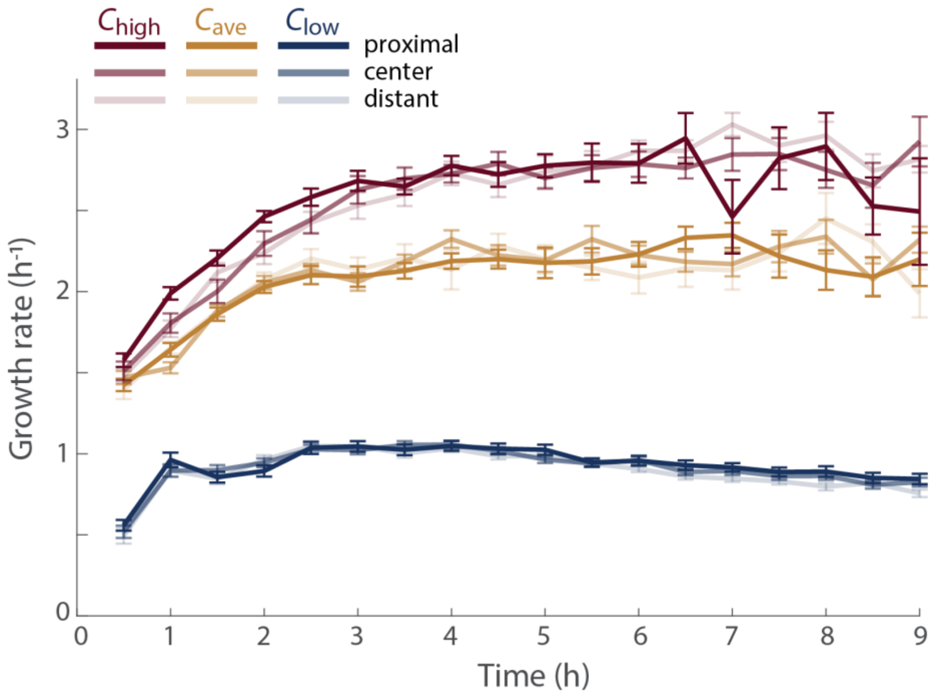

Mean instantaneous growth rate over time from three parallel nutrient concentrations: C_{low} (0.1% LB, blue), C_{ave} (1.05% LB, yellow) and C_{high} (2% LB, red). Cells growing in each concentration were imaged at 10 distinct imaging positions along the microchannel, three of which are shown here represented by lines of different transparency. Proximal refers to the position within the imaging region closest to the signal junction, whereas distant refers to the position furthest from the junction. The lack of systematic variation in growth rate with position suggests that, as expected from the volume of medium present and the flow rate, the nutrient medium was not depleted by cells growing within the microchannel. Error bars denote the standard error of the mean.

Supplementary Fig. 5: Steady-state growth rates under a range of LB dilutions.

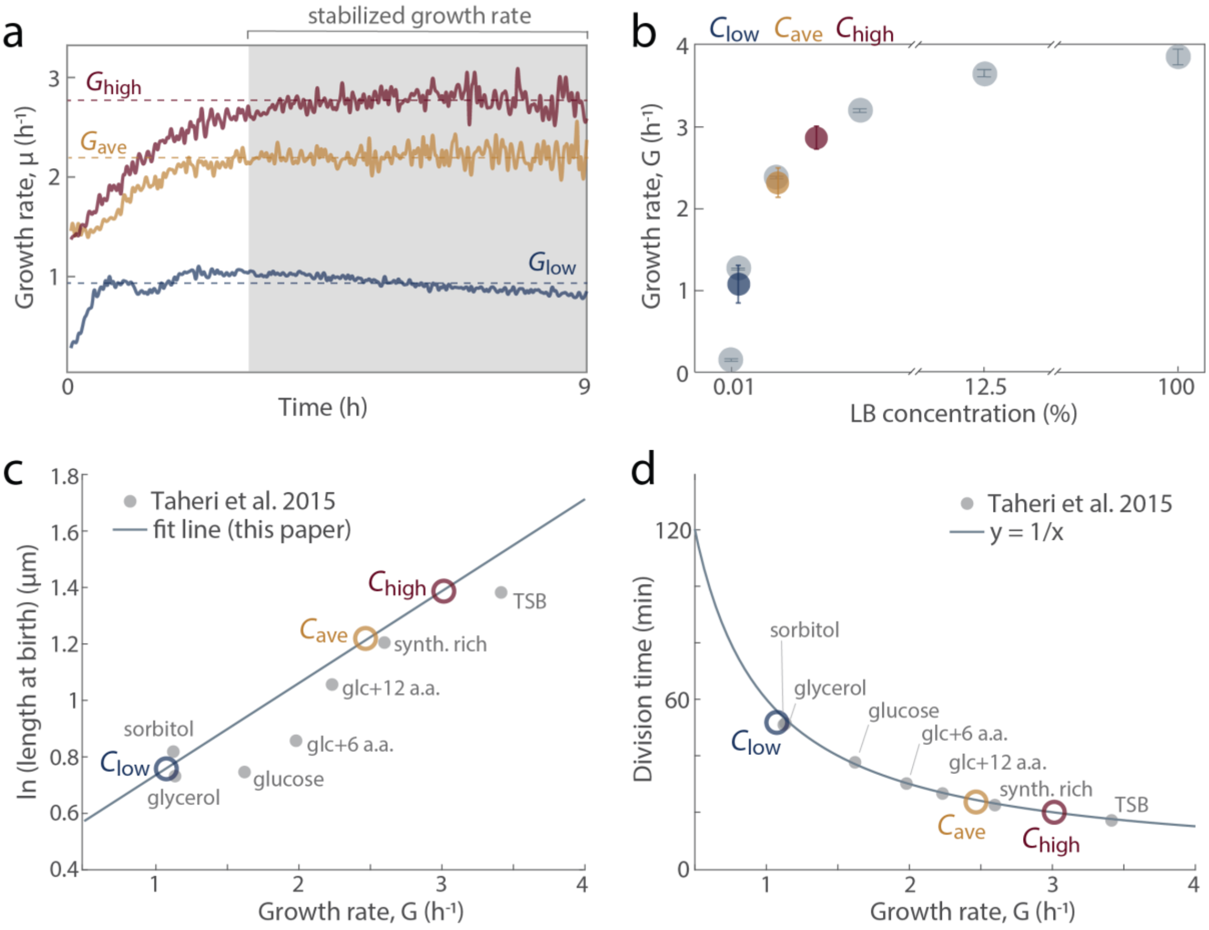

a Growth rate evolving over time in steady C_{low}, C_{ave} and C_{high}. Stabilization of instantaneous growth rate was consistently achieved within the first 3 h of the onset of nutrient delivery. We defined the steady-state growth rate measured from a given condition as the time-average of all instantaneous growth rate data collected after this 3 h mark, indicated by the dashed line and annotated as G_{low}, G_{ave} and G_{high}. **b** Growth rate within our device under various concentrations of LB was empirically characterized from six steady nutrient conditions, ranging from 0.01% to 100% LB. These six steady-state growth rates (gray) – 0.1446 ± 0.0057 (0.01% LB); 1.2622 ± 0.0042 (0.1% LB); 2.3748 ± 0.0057 (1% LB); 3.1924 ± 0.0086 (3.125% LB); 3.6411 ± 0.0170 (12.5% LB); 3.8443 ± 0.0960 (100% LB), all mean ± s.e.m. in units of h^{-1} – were used to reconstruct a Monod curve from which the experimental concentrations were chosen. The final C_{high} (2% LB, red), C_{ave} (1.05% LB, yellow) and C_{low} (0.1% LB, blue) nutrient conditions were chosen such that G_{ave} was clearly distinguishable from G_{low} and G_{high}. Color circles represent the mean steady-state growth rate measured from C_{low}, C_{ave} and C_{high} with error bars representing the standard deviation of the mean among 11–13 replicates (Supplementary Table 2). **c,d** Varying LB concentration reproduces established relationships between growth rate and other cell cycle parameters. Colored points represent mean values measured from conditions in this study, and gray points

represent mean values previously reported from steady conditions in various nutrient media (16) **c** Steady C_{low}, C_{ave} and C_{high} reproduce positive relationship between growth rate and cell size (here, single-cell length at birth). Fit line is drawn from data in this study. **d** Steady C_{low}, C_{ave} and C_{high} reproduce inverse relationship expected between growth rate and division time (the time between cell divisions). Curve is the expected model, not a fit to data.

Supplementary Fig. 6: Nutrient concentration changes in metabolite uptake rates, not the order of metabolite consumption.

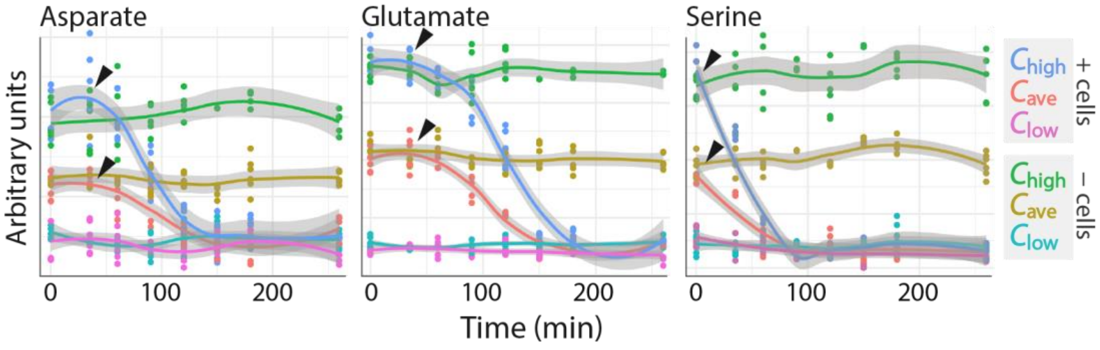

Mass spectrometry analysis of metabolite depletion from batch cultures yielded detection curves for 284 metabolites. Each curve denotes the time course of the detected level of a metabolite in C_{low}, C_{ave} and C_{high} with (+) or without (-) the addition of growing cells at t = 0. Each point indicates the detected value of one biological replicate and the line the mean across replicates (n = at least 3 per time point). Black arrows indicate where metabolite level begins to decrease. The detection curves for aspartate, glutamate and serine are shown here, as they are amongst the first metabolites to be consumed by *E. coli* in complex media (45) and cells in the microchannels effectively experience only the very start of batch culture conditions, as the later stages of batch cultures exhibit changes in medium chemistry due to cell growth and consumption. In our analysis, serine levels begin to deplete after t = 0, and aspartate and glutamate levels begin to deplete after t = 30 min in both C_{ave} and C_{high}. Among the 284 metabolites detected, we never observed a metabolite profile that suggested metabolite depletion occurred at time points that differed between C_{ave} and C_{high}, suggesting that metabolites are consumed in the same order across nutrient conditions. The concentrations of metabolites in C_{low} were below the limit of detection.

Annotations and masses of the 284 detected metabolites. The Kegg annotations of all metabolites detected from the flask cultures of C_{low}, C_{ave} and C_{high} from time-of-flight mass spectrometry are publically available in the Source Data file accompanying this manuscript (http://doi.org/10.5281/zenodo.4697572). These metabolites represent the metabolites from the media not yet consumed by the cultures as well as metabolites secreted into the media by cells. The ion intensity measurements from all 284 detected metabolites across all time points and replicates is available as an Excel file in Source_data titled "metabolomics_intensities.xlsx".

Supplementary Fig. 7: Representative single-cell μ trajectories demonstrate that growth rate dynamics, while noisy, occur in individual cells.

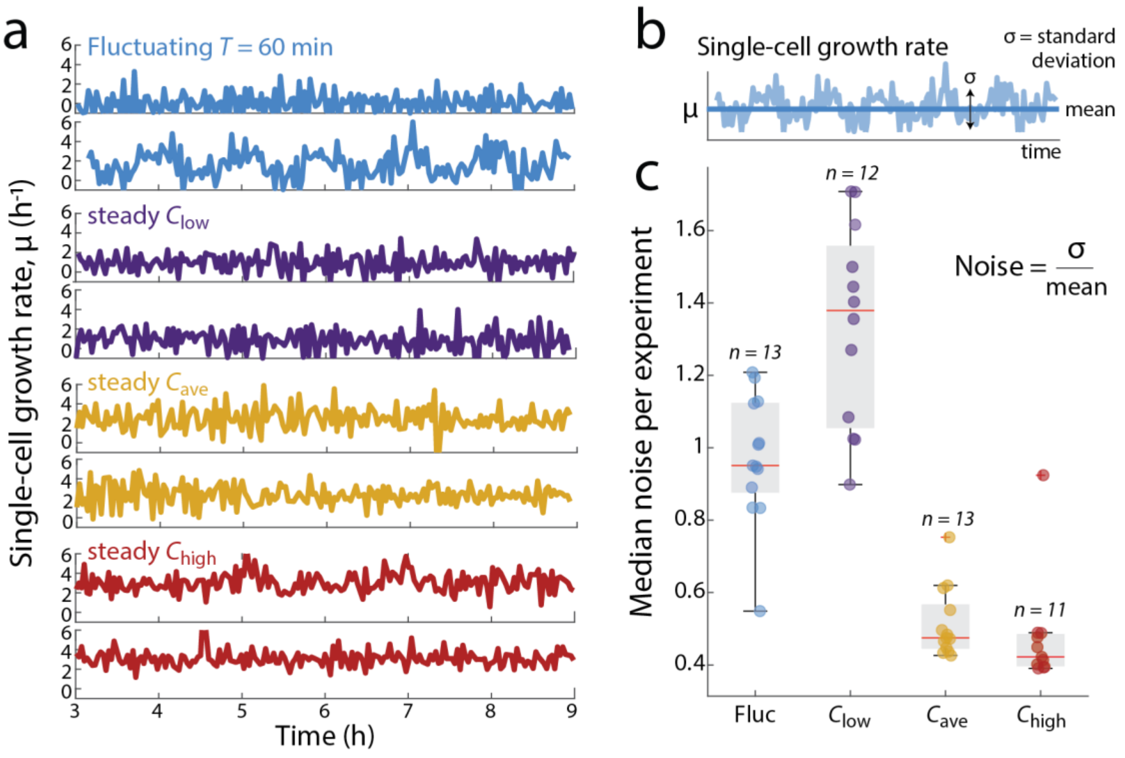

a Visualized instantaneous growth rate dynamics from representative single cell lineages growing in either 60 min nutrient fluctuations (blue) or steady C_{low} (purple), C_{ave} (yellow) and C_{high} (red). For each trajectory, each time step between instantaneous growth rates (μ) was 117 s, with the exception of time steps in which a cell division occurred (leading to a momentary strong negative growth rate), which were omitted for clarity. Due to the high variability in μ measured from time step to time step, growth rate responses to changes in nutrient conditions required averaging across several single cells to detect a significant signal. Still, periodic fluctuations in growth rate are occasionally were occasionally visible by eye from individual trajectories under fluctuating nutrient conditions (second panel, T = 60 min condition). **b** Noise in single-cell growth rate μ was quantified as the standard deviation in μ (σ) over the mean value of μ. Noise was calculated for each single-cell trajectory observed in each experimental condition. **c** Noise in single-cell μ as a function of nutrient condition. Each point represents the median noise value from each biologically independent experimental replicate (n). The median noise measured overall from each condition was 0.95 (fluctuating), 1.38 (steady C_{low}), 0.47 (steady C_{ave}) and 0.42 (steady C_{high}), as indicated by red lines. The minimum and maximum values of each condition were respectively 0.55 and 1.21 (fluctuating), 0.90 and 1.71 (steady C_{low}), 0.43 and 0.75 (steady C_{ave}) and 0.39 and 0.92 (steady C_{high}). The bottom and top edges of the gray box indicate 25th and 75th percentiles, respectively. Whiskers extend to most extreme data points not considered outliers, which are marked by a red plus sign. Noise from the fluctuating condition compiles data from all

fluctuating timescales (T = 30 s, 5 min, 15 min and 60 min). Noise intensity in our data is higher than estimates of inherent biological noise (0.2–0.4) from stochastic fluctuations in metabolism and growth (43). We attribute this increase to measurement noise, primarily derived from fluctuations in measured width, which is smaller and thus noisier than length, and enters squared in the calculation of volume.

Supplementary Fig. 8: Cell division and growth rate in fluctuating nutrient.

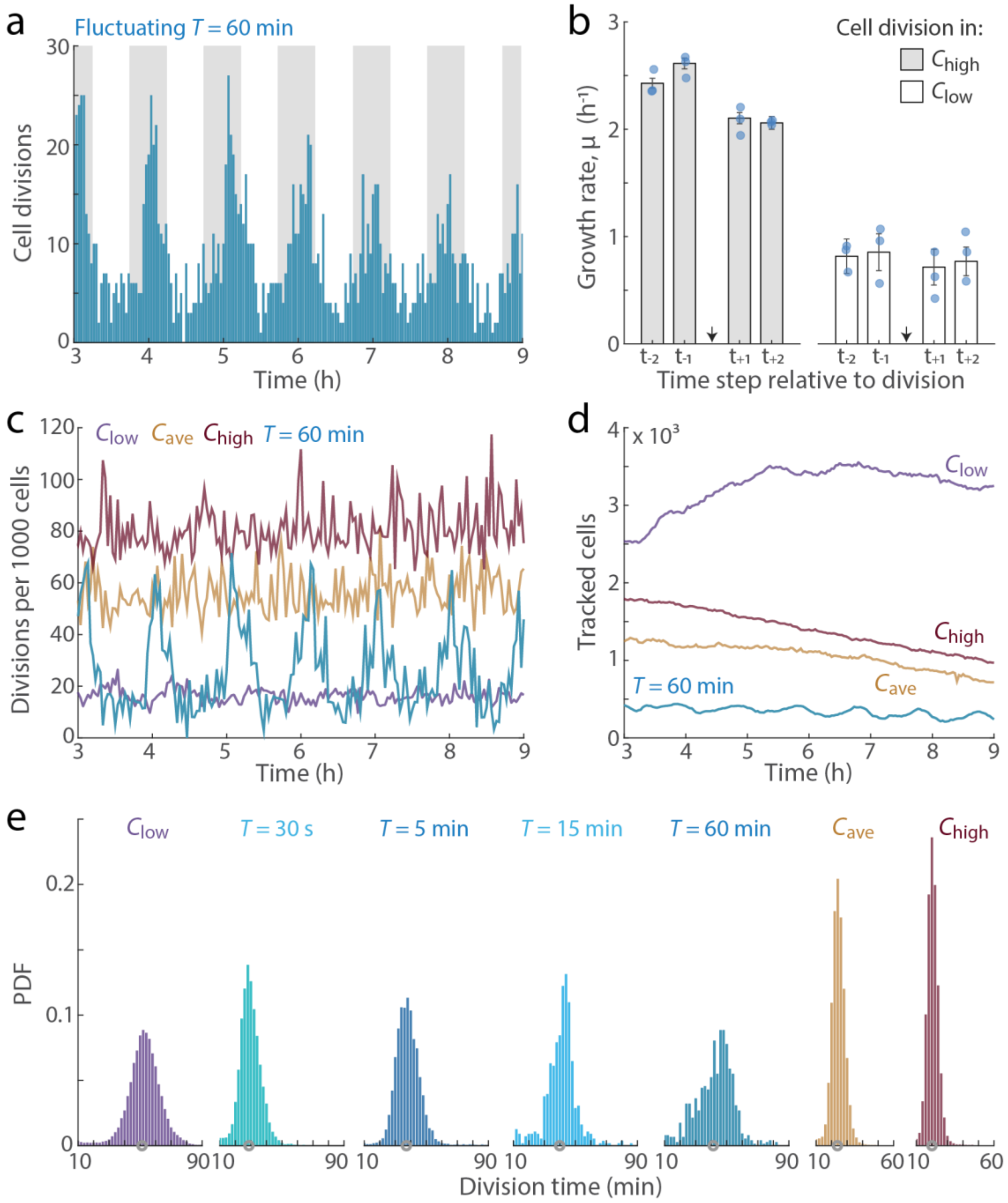

a Cell division events occur more frequently during the high nutrient phases of a fluctuating nutrient signal. Each bar counts the number of cell divisions occurring within each 2 min increment of a fluctuating nutrient condition with a 60 min period. Gray shading indicates phases during which cells experienced C_{high}. **b** Average single-cell growth rate (μ, rate of volume doubling) before and after cell division from single cells grown in 60 min nutrient fluctuations, dividing during either C_{high} or C_{low}. For cells dividing during C_{high}, single-cell growth rate was on average 2.43 ± 0.05 h^{-1} and 2.61 ± 0.05 h^{-1} about 4 min (t_{-2}) and 2 min (t_{-1}) prior to cell division, respectively, and 2.10 ± 0.05 h^{-1} and 2.06 ± 0.06 h^{-1} about 2 min (t_{+1})

and 4 min (t_{+2}) after cell division. For cells dividing during C_{low}, single-cell growth rate was on average 0.82 ± 0.16 h^{-1} and 0.85 ± 0.17 h^{-1} about 4 min (t_{-2}) and 2 min (t_{-1}) prior to cell division, respectively, and 0.72 ± 0.17 h^{-1} and 0.77 ± 0.13 h^{-1} about 2 min (t_{+1}) and 4 min (t_{+2}) after cell division. Each blue point represents the average single-cell growth rate measured from one of three experimental replicates. The value of the bar represents the mean of across replicates; error bars represent standard error of the mean. **c** Production rate of new cells is relatively stable in steady C_{low}, C_{ave}, and C_{high} and fluctuates in T = 60 min nutrient fluctuations. Data shown from one representative experiment of 3 replicates. Total number of tracked cells for this experiment is plotted in **d**. **d** We observed more individual cells in C_{low} than all other conditions. We attribute the different numbers of tracked cells to different physiologies (i.e., surface attachment) promoted by the different conditions. Cell numbers increase in the steady C_{low} condition because cells produced by division can attach to the microchannel and decrease C_{high} since faster growing cells tend to detach more easily. **e** Probability density function (PDF) of division times measured from each nutrient condition in this study. Each PDF contains the division times, the time between consecutive cell divisions from a single-cell lineage, measured from all complete cell cycles tracked after the first 3 h of each experiment (after steady-state growth was achieved). Gray points denote the mean division time of each distribution, which is tabulated below (*n* representing the number of unique cell cycles measured per condition).

Measured division time (min)

	C_{low}	T = 30 s	T = 5 min	T = 15 min	T = 60 min	C_{ave}	C_{high}
mean:	51.3	28.7	37.1	39.9	41.3	23.8	20.0
st dev:	11.7	7.2	8.5	10.2	11.9	4.9	4.4
n:	42,856	12,142	10,093	556	746	32,519	56,639

Supplementary Fig. 9: Frequency of nutrient shifts determine fraction of timesteps containing shifts and change in growth rate after nutrient upshift.

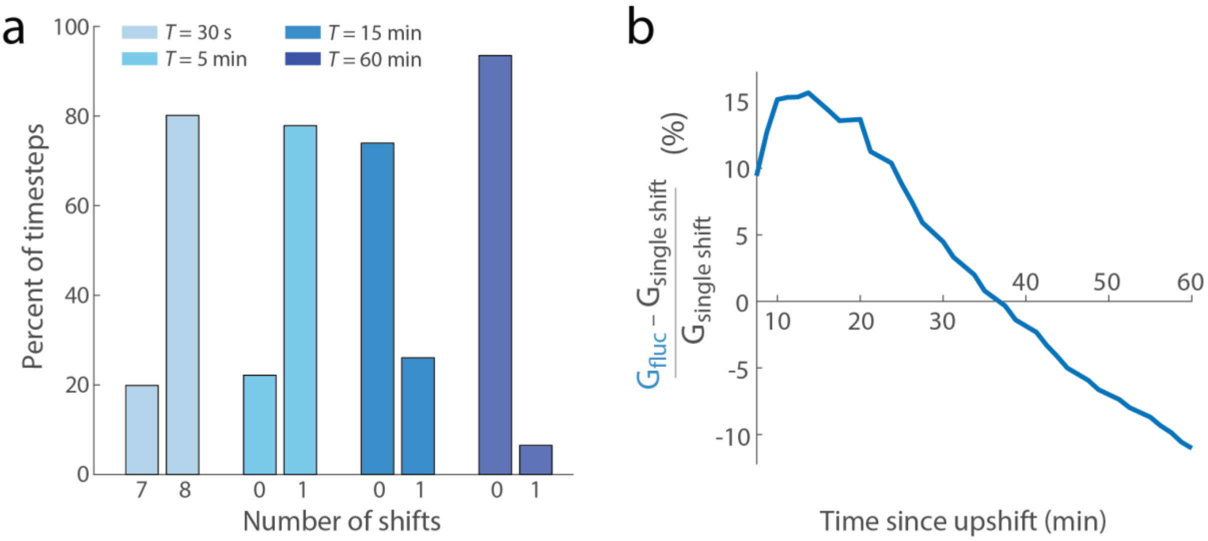

a Percentage of instantaneous growth rate measurements encompassing a nutrient shift for each nutrient signal timescale. A 10 h experiment imaged every 117 s comprises 307 imaging intervals. Given the periods of the nutrient signals delivered (30 s, 5 min, 15 min and 60 min), we calculated the percentage of imaging intervals for each nutrient signal that contained each possible number of nutrient shifts. Each imaging intervals in the fastest nutrient signal (30 s) contains either 7 or 8 nutrient shifts; imaging intervals in longer nutrient signals have either 0 or 1. The decreasing fraction of imaging intervals that contain a nutrient shift with increasing length of the nutrient signal is consistent with the decreasing strength of anticipation, i.e., the phase shift by which growth rate appeared to respond to nutrient shifts before the shift occurred. This calculation is evidence that this phase shift in growth rate response, particularly evident in the 5 min and 15 min nutrient signals (Fig. 2c), resulted from the smoothing of the growth rate signal during analysis, not a biological response in the experiment. **b** Cells adapted to rapid nutrient fluctuations have a growth advantage in the first 30–40 minutes after a nutrient shift. The curve shows the relative difference between the growth rate of fluctuation-adapted cells (*T* = 60 min) and the growth rate of cells grown at steady-state that experience a single nutrient shift as a function of time since a nutrient upshift. The positive value in the first tens of minutes indicates a growth advantage for fluctuation-adapted cells after a nutrient shift. At 30 min after the shift, the growth rate in the fluctuating condition is considered stabilized and used to calculate percent differences from single upshift data extending beyond 30 min (Fig. 4a, b).

Supplementary Fig. 10: Prediction of growth rate dynamics under fluctuations based on the dynamics observed after single shifts.

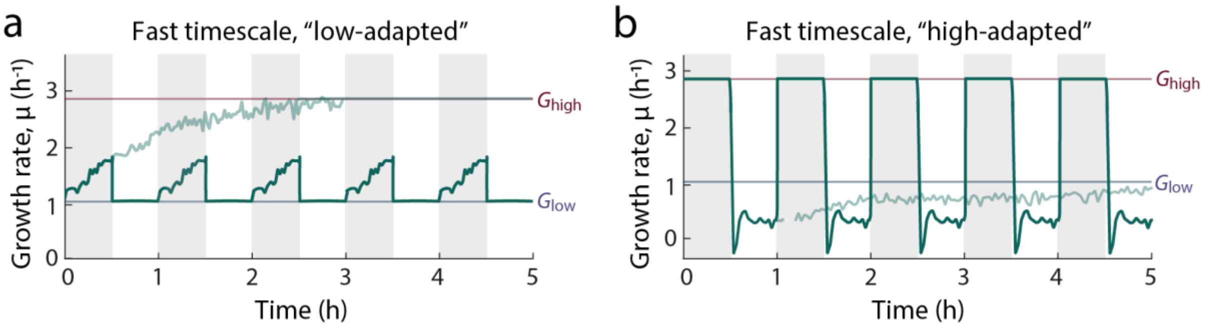

a,b The null model used to predict growth rate dynamics in the absence of a fluctuation-adapted physiology required an assumption under fast fluctuations. We applied the model with two different assumptions: both of are overestimates of growth with only single-shift dynamics due to the cell's immediate ability to return to one steady state. The first assumed that cells were low-adapted, being physiologically adapted for growth at steady-state G_{low}. The second assumed that cells were high-adapted, physiologically adapted for growth at steady-state G_{high}. These assumptions were necessary for nutrient fluctuations on periods of T = 60 min (as exemplified here) or faster, because each nutrient phase (C_{low} or C_{high}) is shorter than the time required for growth rate to transition between steady-state G_{low} and G_{high}. Under both assumptions, the temporal growth rate dynamics are time-averaged to predict G_{fluc} for one full period. **a** After a single nutrient upshift (C_{low} to C_{high}), cells grown in steady C_{low} require 3 h to reach the steady-state growth rate G_{high} (2.86 ± 0.14 h^{-1}). The faded curve is the growth rate response measured after a single upshift (Fig. 4a). The low-adapted null model uses the portion of this single upshift response equal to the duration of the time spent in C_{high}, or 30 min in the plotted example. When the simulated environment returns to C_{low}, the low-adapted growth rate immediately returns to G_{low}. The low-adapted model is the source of the predictions shown in Fig. 6b. **b** After a single nutrient downshift, cells grown in steady C_{high} require at least 5 h to reach the steady-state growth rate G_{low} (1.07 ± 0.23 h^{-1}). The faded curve is the growth rate response measured after a single downshift (Fig. 4a). The high-adapted null model uses the portion of this single downshift response equal to the duration of the time spent in C_{low}, or 30 min in the plotted example. When the simulated environment returns to C_{high}, the low-adapted growth rate immediately returns to G_{high}. All simulated G_{fluc} are displayed in Supplementary Table 5. Gray shading indicates C_{high}.

Supplementary Procedures: Construction of Null Model

Here, we present the null model that we used to compare the mean growth rate, G_{fluc}, measured from fluctuating environments with a mean growth rate predicted from growth rate dynamics lacking the fluctuation-induced physiology. To construct the models, we used the instantaneous growth rate dynamics measured from single nutrient shifts (Fig. 4a), which fundamentally differ from the dynamics of cells grown in fluctuating environments, to simulate growth rate dynamics as if cells continued to shift between steady states.

For long fluctuation periods (T = 12–96 h), each nutrient phase (duration T/2) is sufficiently long for cells to complete the 3–5 h physiological transition to a new steady state before the ensuing shift (Fig. 6a). For T = 12 h, the instantaneous growth rate dynamics were simulated as follows:

1. For the 6 h phase of C_{high}, we took the instantaneous growth rate dynamics measured from the first 3 h after a single upshift. Within 3 h, growth rate reaches and stabilizes at steady-state G_{high}. The remaining 3 h of the 6 h high nutrient phase was simulated as 3 h of steady growth at G_{high} (Fig. 6a).
2. Likewise, for the 6 h phase of C_{low}, we took the instantaneous growth rate dynamics measured from the single downshift experiment at stabilized at steady-state G_{low} 5 h after the shift as the growth rate dynamics for the first 5 h of the low nutrient phase. The remaining 1 h of the 6 h phase was simulated as 1 h of steady growth at G_{low} (Fig. 6a).
3. We averaged the growth rate dynamics simulated across both phases (6 h in C_{high} + 6 h in C_{low}) to calculate the mean growth rate predicted for T = 12 h fluctuations by the null model.

The same approach was used to simulate growth rate dynamics in all longer timescales (i.e., T = 24 h, etc.). The growth rate dynamics in the first 3 h or 5 h (of the C_{high} or C_{low} phase, respectively) remained constant across timescales. After these 3 h or 5 h, growth rate was considered steady at the steady-state growth rate of the current nutrient phase (C_{high} or C_{low}). Thus, the proportion of T spent at steady state increases as T increases, causing the predicted mean growth rate to increase as T increases until the 3-5 h spent adapting to steady state is negligible, at which point the predicted mean growth rate effectively equals G_J (Fig. 6b).

For T shorter than the 3-5 h physiological transition time, we encountered the more complicated situation of predicting what physiology a cell might have once shifted to another nutrient phase. For example, a cell exposed to 30 min of C_{high} cannot reach steady-state G_{high} before the environment switches to 30 min of C_{low}. What physiology does the cell have at the start of C_{low}, and what are the instantaneous growth rate dynamics at the start of the C_{low} nutrient phase?

To avoid complicated and poorly validated assumptions, we chose two simple assumptions that we believe overestimate the predicted mean growth rate from fast fluctuations (T = 30 s – 1 h) in the absence of the fluctuation-induced physiology. Overestimation is acceptable because our conclusions interpret the increase in measured G_{fluc} over G_{fluc} predicted by the null model. Thus, the growth benefit (conferred by the fluctuation-adapted physiology) that we quantify and report is a conservative estimate, due to this overestimation.

We called the two assumptions the low-adapted and the high-adapted version of the null model. The low-adapted model assumed that whenever the cells experience C_{low}, they immediately resume steady growth at G_{low}. Similarly, the high-adapted model assumed that cells, upon experiencing C_{high}, could immediately return to growth at steady G_{high}.

As an explicit example of the construction of these fast timescale simulations, the "low-adapted" model simulating T = 1 h fluctuations are as follows:

1. For the 30 min of C_{high}, we assumed the cells were physiologically at steady-state C_{low}, enabling us to use the first 30 min of the instantaneous growth rate dynamics measured after a single nutrient upshift (Supplementary Fig. 10a).
2. For the 30 min of C_{low}, we assumed the cells were immediately able to grow at steady-state G_{low} and simulated their growth as G_{low} for the entire duration of the low nutrient phase (Supplementary Fig. 10a).
3. As in the slower timescale simulations, we averaged the growth rate dynamics simulated across the entire 1 h period to predict mean growth rate in the null model.

The other fast timescales (T = 30 s, 5 min and 15 min) were simulated in the same manner, only changing the duration of each nutrient phase (T/2). For high-adapted simulations, growth in the high nutrient phase was simulated as steady-state G_{high} and growth in the low nutrient phase simulated as the first T/2 of the instantaneous growth rate dynamics measured after a single nutrient downshift.

We believe the low-adapted and high-adapted versions of the null model overestimate mean growth rate, because in the response to a single shift, time spent in a nutrient condition induces a physiological transition that moves the cell away from the steady-state physiology of the previous conditions. Thus, assuming an immediate return to steady state is an upper bound, and therefore a conservative prediction when considering that we conclude that the measured growth rate in second- to minute-scale fluctuations is greater than predicted (Fig. 6b).

Supplementary Table 1

Mean lag times between signal generation and signal delivery to cells, per experiment.

Lag time was calculated from the flow rate and effective cross-sectional area. Error denotes the standard deviation of the 10 cell positions sampled per experiment, across the length of the imaging region. Each straight channel delivers fluid from one steady source, a 10 mL syringe loaded into the same Harvard Apparatus syringe pump, which pushes the fluid into the channels at a flow rate of 15 μL min^{-1}. The different flow rates used (15–27 μL min^{-1}) did not affect growth rate (Supplementary Fig. 2a).

Experimental condition	Flow rate, Q (μL min^{-1})	Lag time (s)
30 s period (2017-11-12)	15	2.94 ± 0.12
30 s period (2017-11-14)	21	2.08 ± 0.08
30 s period (2018-01-04)	27	1.63 ± 0.06
5 min period (2017-10-10)	Not recorded	N.A. (assigned lag time = 1.7 s)
5 min period (2017-11-15)	21	2.08 ± 0.08
5 min period (2018-01-11)	21	2.07 ± 0.07
15 min period (2017-11-13)	20	2.20 ± 0.08
15 min period (2018-01-12)	22	1.97 ± 0.07
15 min period (2018-01-16)	22	1.98 ± 0.07
15 min period (2018-01-17)	23	1.89 ± 0.07
60 min period (2018-01-29)	26	1.67 ± 0.05
60 min period (2018-01-31)	26	1.69 ± 0.07
60 min period (2018-02-01)	26	1.70 ± 0.07

Supplementary Table 2

Summary table of steady-state growth rates, averaged by nutrient condition. Mean value of *G* of replicates for each condition, reported with standard deviation (st dev), standard error of the mean (s.e.m.) and number (*n*) of experimental replicates for each condition. The values measured from each experimental replicate are listed in Supplementary Table 6. The mean division time for each condition predicted from the inverse of these growth rates (*G*) is shown alongside the measured division time from each condition, for comparison. Measured division time was measured from individual cell cycles (*N*) compiled across all experimental replicates (*n*).

	C_{low}	C_{ave}	C_{high}	T = 30 s	T = 5 min	T = 15 min	T = 60 min
mean:	1.0694	2.3094	2.8567	1.9331	1.5319	1.1664	1.1474
st dev:	0.2271	0.1768	0.1363	0.1644	0.1998	0.3030	0.1354
s.e.m.:	0.0656	0.0490	0.0411	0.0949	0.1154	0.1515	0.0782
n:	12	13	11	3	3	4	3

Supplementary Table 3

Predicted differences in biovolume production across fluctuation timescales, relative to the steady average environment. The mean growth rate measured from each fluctuating environment, G_{fluc}, was used to calculate the daily biovolume (daily $M(t)$) expected to be generated per 1 μm^3 cell. Relative to the daily biovolume expected to be produced at the mean growth rate under the steady average conditions, G_{ave}, cells in the fluctuating environments are predicted to produce 10^2–10^8 times less biovolume per day.

Growth condition (G)	Mean G (h^{-1})	Predicted daily $M(t)$	Fold difference, $M(t)/M(t)_{\text{ave}}$
Steady average (G_{ave})	2.31	5×10^{16}	1
T = 30 s ($G_{\text{fluc},30}$)	1.93	9×10^{13}	5×10^{2}
T = 5 min ($G_{\text{fluc},5}$)	1.53	1×10^{11}	5×10^{5}
T = 15 min ($G_{\text{fluc},15}$)	1.15	2×10^{8}	2×10^{8}
T = 60 min ($G_{\text{fluc},60}$)	1.15	2×10^{8}	2×10^{8}

Supplementary Table 4

Stabilized growth rate in fluctuations as a percentage of steady-state growth rate. Under fluctuating nutrient environments, growth rates stabilize within 2–3 min of a nutrient shift. The second column quantifies the mean stabilized growth rate and standard deviation between replicates (*n* = 3 or 4). These stabilized growth rates are considerably lower than the steady-state rate corresponding to the post-shift environment: G_{high} (2.86 ± 0.14 h^{-1}) in the case of upshifts, G_{low} (1.07 ± 0.23 h^{-1}) in the case of downshifts. The reduction in growth rate relative to the "target" steady-state is quantified in column three (% loss = $\frac{G_{steady} - G_{fluc}}{G_{steady}} \cdot 100$).

	Fluctuation timescale	Stabilized growth rate (h^{-1})	% loss from steady-state
Upshift	15 min	1.86 ± 0.47	35.0 ± 0.2
	60 min	1.86 ± 0.13	35.0 ± 0.1
Downshift	15 min	0.65 ± 0.22	39.3 ± 0.3
	60min	0.60 ± 0.10	43.9 ± 0.3

Supplementary Table 5

Predictions of G_{fluc} based on the growth rate dynamics observed after single nutrient shifts. Values are calculated based on measured single-shift or steady-state data by one of three methods: slow for long timescales of nutrient shifts (T = 12 h and greater), and low-adapted or high-adapted for faster nutrient timescales (Supplementary Figure 9). Both low-adapted and high-adapted null models predicted lower than measured values of G_{fluc} for fast nutrient fluctuations. Both models also predicted lower G_{fluc} with decreasing nutrient timescale (T), whereas the opposite trend was observed from the measured G_{fluc}. All predicted values of G_{fluc} in this Table are reported as a fraction of G_{ave} (2.31 h^{-1}).

Timescale (T)	Slow	Low-adapted	High-adapted
96 h	0.830	N.A.	N.A.
48 h	0.810	N.A.	N.A.
24 h	0.769	N.A.	N.A.
12 h	0.688	N.A.	N.A.
60 min	N.A.	0.563	0.644
15 min	N.A.	0.511	0.638
5 min	N.A.	0.502	0.623
30 s	N.A.	0.497	0.619

Supplementary Table 6

Summary table of steady-state growth rates measured from each experimental replicate. Mean growth rate (*G*) and standard error of the mean for all conditions from all replicate fluctuating experiments. Cells labeled with N.A. represent instances in which a bubble formed within the channel during the first 3 h of data acquisition, preventing the determination of a steady-state growth rate. Units of growth rate are h^{-1}. The column labeled "Experiment" lists the date on which the experiment was performed. Each row associated with one fluctuation timescale represents a distinct experimental replicate carried out simultaneously in one microfluidic device.

	Experiment	Fluctuating	Steady C_{low}	Steady C_{ave}	Steady C_{high}
30 s	2017-11-12	1.8391 ± 0.0040	1.0965 ± 0.0024	2.4439 ± 0.0053	2.9497 ± 0.0067
	2017-11-14	2.1229 ± 0.0040	1.2170 ± 0.0038	2.4692 ± 0.0087	2.6744 ± 0.0183
	2018-01-04	1.8373 ± 0.0077	0.8430 ± 0.0036	2.2755 ± 0.0092	2.9907 ± 0.0109
5 min	2017-10-10	1.6728 ± 0.0063	1.4814 ± 0.0050	2.6296 ± 0.0105	2.8919 ± 0.0143
	2017-11-15	1.6198 ± 0.0075	1.3525 ± 0.0054	2.3198 ± 0.0098	2.9588 ± 0.0110
	2018-01-11	1.3032 ± 0.0037	0.9503 ± 0.0018	2.1744 ± 0.0088	N.A.
15 min	2017-11-13	1.5064 ± 0.0109	1.3282 ± 0.0061	2.4434 ± 0.0105	3.1206 ± 0.0150
	2018-01-12	1.3302 ± 0.0077	N.A.	2.4605 ± 0.0083	N.A.
	2018-01-16	0.9695 ± 0.0123	0.9870 ± 0.0024	2.0733 ± 0.0151	2.7382 ± 0.0156
	2018-01-17	0.8595 ± 0.0123	1.0341 ± 0.0029	2.0362 ± 0.0141	2.7467 ± 0.0092
60 min	2018-01-29	1.2941 ± 0.0096	0.7944 ± 0.0026	2.3412 ± 0.0055	2.8273 ± 0.0050
	2018-01-31	1.1211 ± 0.0129	0.8357 ± 0.0026	2.1422 ± 0.0063	2.7684 ± 0.0096
	2018-02-01	1.0272 ± 0.0091	0.9130 ± 0.0022	2.2123 ± 0.0076	2.7572 ± 0.0074

Supplementary Table 7

Daily correlations between the time-averaged growth rate (*G*) of different nutrient conditions. The correlation coefficient, *r*, was measured for each pair of steady conditions, by fitting a linear regression to *G* measured from parallel channels (i.e. replicates conducted on the same day, on the same microfluidic chip and seeded simultaneously with the same starting culture). These moderate correlations between conditions performed on the same day indicate that slight differences between the seed culture contributed variability in growth rate measured from identical conditions performed on different days. Thus, we compared growth rate across conditions performed on the same day before comparing experimental replicates.

	G_{low}	G_{ave}	G_{high}
G_{low}	1		
G_{ave}	0.662	1	
G_{high}	0.359	0.434	1
