# Supplementary material for: A distinct growth physiology enhances bacterial growth under rapid nutrient fluctuations
Source: Nat Commun. 2021 Jun 16;12:3662. doi: 10.1038/s41467-021-23439-8 (PMC8209047; doi:10.1038/s41467-021-23439-8)
Supplement: Supplementary file 3 — Reporting Summary [file 41467_2021_23439_MOESM3_ESM.pdf]

nature research

Corresponding author(s): Jen Nguyen; Roman Stocker

Last updated by author(s): Apr 16, 2021

Reporting Summary

Nature Research wishes to improve the reproducibility of the work that we publish. This form provides structure for consistency and transparency in reporting. For further information on Nature Research policies, see our Editorial Policies and the Editorial Policy Checklist.

Statistics

For all statistical analyses, confirm that the following items are present in the figure legend, table legend, main text, or Methods section.

n/a	Confirmed	
☐	☒	The exact sample size (*n*) for each experimental group/condition, given as a discrete number and unit of measurement
☐	☒	A statement on whether measurements were taken from distinct samples or whether the same sample was measured repeatedly
☒	☐	The statistical test(s) used AND whether they are one- or two-sided<br>*Only common tests should be described solely by name; describe more complex techniques in the Methods section.*
☒	☐	A description of all covariates tested
☒	☐	A description of any assumptions or corrections, such as tests of normality and adjustment for multiple comparisons
☐	☒	A full description of the statistical parameters including central tendency (e.g. means) or other basic estimates (e.g. regression coefficient) AND variation (e.g. standard deviation) or associated estimates of uncertainty (e.g. confidence intervals)
☒	☐	For null hypothesis testing, the test statistic (e.g. *F*, *t*, *r*) with confidence intervals, effect sizes, degrees of freedom and *P* value noted<br>*Give P values as exact values whenever suitable.*
☒	☐	For Bayesian analysis, information on the choice of priors and Markov chain Monte Carlo settings
☒	☐	For hierarchical and complex designs, identification of the appropriate level for tests and full reporting of outcomes
☒	☐	Estimates of effect sizes (e.g. Cohen's *d*, Pearson's *r*), indicating how they were calculated

Our web collection on statistics for biologists contains articles on many of the points above.

Software and code

Policy information about availability of computer code

Data collection	Commercial software: MATLAB (R2019), NIS-Elements (version 4.6). Custom code for data collection is openly documented on Github at https://github.com/jkimthu/under-pressure and published on Zenodo at http://doi.org/10.5281/zenodo.4646684 (v1.0.0).
Data analysis	Commercial software: MATLAB (R2019). Open source software: Fiji (version 2.1.0). Custom code for data analysis is openly documented on Github at https://github.com/jkimthu/growing-up and published on Zenodo at http://doi.org/10.5281/zenodo.4646499 (v1.0.0).

For manuscripts utilizing custom algorithms or software that are central to the research but not yet described in published literature, software must be made available to editors and reviewers. We strongly encourage code deposition in a community repository (e.g. GitHub). See the Nature Research guidelines for submitting code & software for further information.

Data

Policy information about availability of data

All manuscripts must include a data availability statement. This statement should provide the following information, where applicable:

- Accession codes, unique identifiers, or web links for publicly available datasets
- A list of figures that have associated raw data
- A description of any restrictions on data availability

Raw mass spectral data is deposited to massIVE and accessible with the accession code MSV000087096. The KEGG database used to annotate mass spectral data is available at https://www.genome.jp/kegg/pathway.html restricted to organism eco. Raw image data that support this study are 104 GB and available from the corresponding authors upon request. Datasets of measurements from raw images are the Source data of this manuscript are available at http://doi.org/10.5281/zenodo.4697572 (50). The Source Data can be directly input into the openly available source code to produce reported figures and calculations (see Code Availability).

Field-specific reporting

Please select the one below that is the best fit for your research. If you are not sure, read the appropriate sections before making your selection.

☒ Life sciences ☐ Behavioural & social sciences ☐ Ecological, evolutionary & environmental sciences

For a reference copy of the document with all sections, see nature.com/documents/nr-reporting-summary-flat.pdf

Life sciences study design

All studies must disclose on these points even when the disclosure is negative.

Sample size	Legend of Fig. 2 notes the minimum number of cells (n=1842) from which growth rate was quantified per replicate experiment. The sample size was not determined prior to study, as cell-to-cell variability was unknown (especially under nutrient fluctuations). Thus, we used the maximum sample size feasible from our newly developed experimental method.
Data exclusions	Timepoints in which imaging data went out of focus were excluded from analysis. This loss in focus pertained only to a few minutes in a couple of experiments, specifically the single nutrient shift experiments, such as those plotted in Fig. 4a and 4c. In these plots, the data excluded are observable as a gap in the data (growth rate vs. time). Within this gap, we visually confirmed from raw image data when focus was lost and regained, and removed these time points from analysis to avoid erroneous values from being interpreted as biological behavior. The exact experimental datasets (as shared in Source_data) and time points excluded are identified in the open access code published in the Github repository https://github.com/jkimthu/growing-up.
Replication	All experimental replicates in this study refer to biological replicates, which were performed on different days with different seed cultures and media preparations. The number of replicates for each condition are reported in Figure Legends and statistics for each replicate are tabulated in Supplementary Table 7.
Randomization	Within a single experiment, clonal cells from the same bacterial culture were used to seed four parallel microfluidic channels, which each delivered a different experimental nutrient concentration. Thus, the individual E. coli cells observed in each of the four conditions were randomly selected from as uniform a cell population as experimentally possible.
Blinding	Genetically (clonal) and phenotypically (same culture) cells were randomly allocated for each experimental condition. Selection bias was thus not a relevant issue to this study, as it was important to us that cells in each condition were identical apart from the environmental conditions they were exposed to after allocation.

Reporting for specific materials, systems and methods

We require information from authors about some types of materials, experimental systems and methods used in many studies. Here, indicate whether each material, system or method listed is relevant to your study. If you are not sure if a list item applies to your research, read the appropriate section before selecting a response.

Materials & experimental systems

n/a	Involved in the study
☒	☐ Antibodies
☒	☐ Eukaryotic cell lines
☒	☐ Palaeontology and archaeology
☒	☐ Animals and other organisms
☒	☐ Human research participants
☒	☐ Clinical data
☒	☐ Dual use research of concern

Methods

n/a	Involved in the study
☒	☐ ChIP-seq
☒	☐ Flow cytometry
☒	☐ MRI-based neuroimaging
